# Relationship Between Unhappy Musicians, Resistance Toward Innovation and Uncreative Music Products: Psychological Security as Moderator

**DOI:** 10.3389/fpsyg.2022.922404

**Published:** 2022-06-15

**Authors:** Wei Liang, Shamim Akhter, Tribhuwan Kumar

**Affiliations:** ^1^Chinese Instruments Department, Zhejiang Conservatory of Music, Hangzhou, China; ^2^School of Languages, Civilisation and Philosophy, Universiti Utara Malaysia, Changlun, Malaysia; ^3^College of Science and Humanities at Sulail, Prince Sattam Bin Abdulaziz University, Al-Kharj, Saudi Arabia

**Keywords:** unhappy musicians, resistance to innovation, uncreative music products, psychological security, Malaysia, music learning institutions, smart-PLS

## Abstract

Recently, uncreative music products have become a global issue due to the unhappy musicians and resistance to innovation that needs researchers’ focus. This article explores the impact of unhappy musicians and resistance to innovation on uncreative music products in Malaysia. This article also investigates the moderating role of psychological security among the relationships of unhappy musicians, resistance to innovation, and uncreative music products in Malaysia. This study has applied the questionnaire method to gather the primary data from the selected respondents. The researchers have also applied the smart-PLS to check the nexus among constructs and test the hypotheses. The results revealed that unhappy musicians and resistance to innovation have a significant and positive linkage with uncreative music products in Malaysia. The results also revealed that psychological security significantly moderates the linkage among unhappy musicians, resistance to innovation, and uncreative music products in Malaysia. Thus, this study guides the regulators to develop the regulations to reduce the unhappiness among musicians and motivates the regulators to adopt innovation to increase the creative music product in Malaysia.

## Introduction

Over the past few decades, the world is witnessing rapid change as a result of globalization. There are a number of industries operating in a country. Some work to support the economy, whereas the others work for the society. Both prevail in their unique importance. Like many other industries, the music industry of the world also changed at a rapid pace. Globalization has also expressed its impact on this industry (Jansson, 2020; Anyanwu and Sylvanus, 2022). Music has become an essential part of society. Another reason for evolving of this industry is cultural exchange. The cultural exchange has also enhanced the importance of this industry (Fosler-Lussier, 2012). Earlier, the music industry was not accepted as a carrier but now it has become one of the fastest-growing reasonable sources of society’s earnings. There are a number of factors that impact the music industry like country norms and values, the society’s acceptance of music, religious factors, and government support. In the past, the music was designed for the national level only, but as the result of cultural exchange, this industry has also crossed the borders and introduced its flavor to the other country. This has enabled the music industry professionals to introduce some differences in order to attract the national and international audiences. The world has become above typical national music. Rapid changes are witnessed with every hour. This industry has also become a good financial supporter of the economy. As the world has become a global village, it also strongly impacted the music industry. The music-related firms or individuals also try to introduce some innovations in order to secure a competitive advantage (Xing, 2018). The music individual who failed to modify their music as per society’s requirements is crushed by the high volume of competition.

Malaysia is one of the fastest-growing economies. The music industry of Malaysia is also getting reasonable attention from the world. As the local commercial music business has gained importance in Malaysian society, the country has become richer and more economically developed. Although Malaysian popular music nowadays closely follows the footprints and patterns of Western and International popular streams, the tendency does indicate an individual sense of artistic expression in modern Malaysian society (Hamzah and Johan, 2020). Furthermore, during the last 15 years, the number of radio stations and television channels airing a diverse range of music has increased dramatically. However, in recent years, the country has witnessed the emergence of a fresh and experimental indigenous music scene that has grown faster than ever to become a force to be reckoned with in the worldwide music arena. This is despite the fact that Malaysian anthropometry music has a very short history and that the country’s Internet user saturation rate has only recently increased. Malaysia is a mixed country with a wide musical heritage. Malaysian music may have edgy marketability and adaptability, as each ethnic group plays its own traditional music while also sharing the diversity of music. The following issues related to this industry urged to conduct this study: (1) inadequate music education, (2) lack of government support, (3) lack of resources and teaching staff, (4) need for music syllabus revamp, (5) decline in the recording industry, (6) lack of talent development programs (Hamzah and Johan, 2020), (7) lack of mentoring programs, (8) lack of financial support of talented musicians and artists, and (9) lack of proper composer rights (Alavi and Azmi, 2019).

This study addresses some gaps that exist in the literature: (1) Being one of the important and liked concept studied, music still has not reached its peak; (2) Sitonio and Nucciarelli (2018) investigated whether block-chain has any impact on music industry, whereas this study tests the association between musicians and music products with the addition of moderation impact in Malaysian music industry; (3) Pelloquin (2020) worked on music industry-related career advancement barrier, whereas this tested the association between musicians and music products with the addition of other variables like resistance toward innovation and psychological insecurity in Malaysian music industry; (4) checked whether the music industry can be a part of colleges’ music-related programs, whereas this study tests the association between musicians and music products with the addition of other variables like resistance toward innovation and psychological insecurity in Malaysian music industry; (5) the model is not tested before in Malaysia, hence this study checks the model in a Malaysian perspective with new data set; (6) Chen et al. (2022) worked on the music products’ marketing strategy, whereas this study works on the music products’ creations in Malaysia with the addition of moderation effect. The significance of the study is as follows: (1) it highlights the importance of the music industry in Malaysia which is not at its peak; (2) it helps music industry-related academicians and professionals revamp their policies to support for the betterment of the music industry in Malaysia; (3) it helps the researchers to identify the importance of the music-related product for any country like Malaysia; (4) it provides the importance of innovation adoption in the music industry, while resistance to innovation reduces the creative music products; and (5) it helps the new researchers in examining this area in the future.

The study structure is divided into five phases. The first phase presents the introduction. In the second phase of the study, the pieces of evidence regarding unhappy musicians, resistance toward innovation, psychological insecurity, and uncreative music products are discussed in the light of past literature. The third phase of the study shines the spotlight on the methodology employed for the collection of data regarding unhappy musicians, resistance toward innovation, psychological insecurity, and uncreative music products, and its validity is analyzed. In the fourth phase, the results of the study are compared with the pieces of evidence reviewed from the literature. In the last phase, the study implications along with the conclusion and future recommendations are presented, which concludes this study.

## Literature Review

Many countries offer pleasant careers to innovative people who are beneficial in nourishing the name of their respective countries (Chien et al., 2021b). Musicians are considered a renowned career in the United States where the happiness of musicians could not be measured. In Malaysia, the young and fresh musicians have attained much importance in the world, which is compulsory to be known by the people visiting Malaysia. Rasmussen (2021) examined the student cohorts with supportive and formidable communities that are important for influencing uncreative music products. This also leads to the uncreative music that is produced by the musicians who are unhappy due to wasting their career if their earnings are less than their spending. Wicker (2020) analyzed the impacts of travel and the frequency of participation of musicians that leads to the uplifting of tourists’ minds. Currently, participation in sports is inclusive of music, and the unhappy commuter of music could probably impact the products of music. Gupta (2019) emphasized on certain online channels like Amazon, which has taken the fleet of musicians and removed the unhappiness of musicians. This significantly helped the innovation and creativeness of music products increasingly sold on online channels. When there is any unfairness toward the music product, there is ultimately the mood and acts of musicians behind them. Avis (2020) enumerated that the higher education of music students in many countries like Malaysia increases the creativeness of music products. The unhappy musicians are mostly the main reason living in Malaysia that impacts the music products. In the world, musicians are actually considered as unhappy when they are maltreated by the public and hear abusive comments. Rather than motivating the musicians who are actually relaxing the minds and hearts of people with creative products, they are being victims due to the miserable attitude of people. Devroop and Titlestad (2021) investigated the levels where the arts meet science and whether the relationship between music and signification exists. Ultimately, the science meeting arts provides unlimited satisfaction to the people of different moods, and music is one of the best arts that are helping the musicians with creative music products. In Malaysia, the rating of musicians is relatively higher for musicians who are young irrespective of those who are only singing in the bars and considered uncreative for music products.

**H1:** Unhappy musicians significantly influence uncreative music products.

Over the past few decades, innovation in many countries all over the world is resisted by many industries, especially in Malaysia. This is due to the consistency of working by the old people who are satisfied with their careers without technology (Chien et al., 2021a). The old people are not fond of new music, and they are being the main resister influencing the uncreative music products. Stryja and Satzger (2019) investigated the impacts and involvement of digital nudging in overcoming the resistance toward innovation. Therefore, the decisions of some old and other people have been changed with the digital music that impacted the minds and hearts of people in Malaysia. The digital use of music and innovation in music are termed as the nominated trend for the rise and importance of music in Malaysia. van der Merwe et al. (2020) explored the dynamics and functions of innovation that are resisted by the health projects and the involvement of certain communities. From the various communities, the resistance toward the innovation in music has been considered violent. Some *status quo* people are breaking the potential among young musicians and are the main resistance toward innovation. Schartinger et al. (2020) narrated the importance of green social innovation toward the typology of better music products. The violence in innovation has narrated a negative impact on the products of music and leads to the lack of schooling for musicians. The prevailing conflicts of resistance and lack of beliefs in the structuring of music products lead to minimizing innovation. Bennett (2020) analyzed the connection between the music industry, digitalization, administration and non-creativeness in embedded working. Sometimes, the people, as well as consumers, show their unwillingness to adapt to the change, services, and products. There is resistance from parents toward the innovation in music, which is highly influential for the uncreative music products. Xiao (2021) examined the introduction of digital media and the eradication of resistance to the innovation that increased the creative music products in the market. In Malaysia, the music has attained significant importance due to the rise of some young and new musicians, and this is only due to the consistency in innovation. These musicians have taken the music industry at significant stages, and new bread is also supporting the evident value of innovation in this music.

**H2:** Resistance toward innovation significantly influences uncreative music products.

Among career musicians, a certain level of psychological insecurity leads to numerous factors. These factors comprise the uncertainty, inadequacy and anxiety that impact the moods of musicians and creativeness in products. Ugwu et al. (2021) examined the relationship between psychological wellbeing and insecurity of roles, and the positive expectations among unhappy musicians and uncreative music products must be resolved. When there is an insecurity of psychology in the musicians, the ultimate impact exists on the unhappiness of musicians and uncreative products. Ramachandran and Vertinsky (2020) assessed the musical arts with the relationship between dances, ragas, movement and music with significant orientations. Many organizations work for the upgradation of musical products, and this could only be attained by eradicating the elements of psychological insecurity. Some musicians are unhappy in Malaysia due to the lack of importance toward the music arts, and this contributes to uncreative music products. These uncreative products in the market not only damage the art and music industry but also pose an unhappy attitude for the musicians. It is important to set the proper limits among the people who could help in achieving realistic goals. These goals have been significantly implemented in Malaysia while viewing the deliberate focus on psychological insecurity. Eisenberg (2019) investigated the politics of language and music that is improvised due to the bunch of riffs in the communities. These define the constant evaluation of musical fragment transforms that can uplift the musical products. Insecurity not only leads to mental illness but also disrupts the relationships among the musicians and the communities. There are standards set by the people for the music, and the existence of psychological insecurity inserts an uncertain impact on the uncreative music products. In Malaysia, the causes of insecurity are linked with societal expectations that are associated with the happiness and unhappiness of musicians. Hattangadi et al. (2021) analyzed the association of insecurity of psychological attitudes that distress the quality of education of students. It creates different elements of anxiety, pressure, and depression over the musicians who influence the uncreative music products. While attaining the goals and objectives in the musical products, there is a deem need of creating happiness for the musicians. To achieve the objectives of significant and dominant uncreative music products, there is a need of eliminating psychological insecurity.

**H3:** Psychological insecurity significantly moderates between unhappy musicians and uncreative music products.

The standards set by the people who are interacted most like family, friends, and social networks cause psychological insecurity. Due to this, the effect of psychological insecurity is clear between the creativeness in music products and resistance to innovation prominently in Malaysia. Kim-Mozeleski et al. (2021) examined the reciprocal impacts of distress and insecurity on psychological attitudes toward innovation and musical products. The psychological distress has been placing independent risks not only in the field of food and smoking but also in other fields. The feelings of security are considered a syndrome for the people and the increasing trend of innovation. Tavares Furtado (2020) assessed the values of resistance toward the innovation that is posing the dominant trend toward the uncreative music products. This is widely supported by the moderating role of psychological insecurity that interprets the uncreative music products and resistance to innovation. The rising narrative of psychological insecurity requires elimination for the proper diffusion of resistance toward innovation and uncreative music products. Therefore, the resistance to the innovation inserts a significant impact on the uncreative music products; however, the psychological insecurity also poses moderating impact. Mihaylova (2020) investigated the illusion, television, and radical performance of art fields that are progressed by the elimination of insecurity in psychology. In the fields of music, the growing innovation and technology has not only improved the sound system of music products but also lead to strong psychology. Especially in the musical sector of Malaysia, the psychological insecurity must be resolved, which could not only promote innovation but also promote music products. Ciciurkaite and Brown (2018) enumerated the factors of psychological insecurity that are associated with alcohol use, distress, and insecurity of food. These factors are silent in the industry of music, which is creating disparity among the communities and posing a negative impact. The unstable security of psychology is responsible for the resistance toward innovation and uncreative music products. There is a diversification of attitudes in people who mostly constitute the feeling of insecurity that uplifts the resistance toward innovation. Mostly in Malaysia, the segregation of gender attitudes is considered a depressive symptom of the resistance toward innovation and uncreative music products.

**H4:** Psychological insecurity significantly moderates between resistance toward innovation and uncreative music products.

## Materials and Methods

**FIGURE 1:**
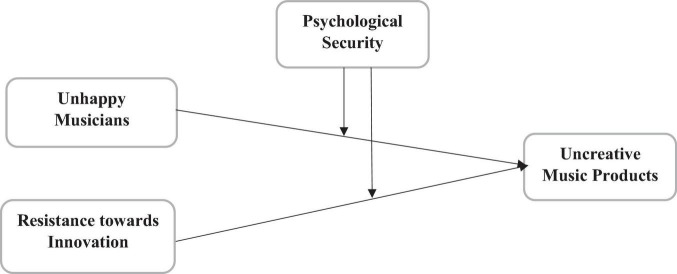
Theoretical model.

This article explores the impact of unhappy musicians and resistance to innovation on uncreative music products in Malaysia. It also investigates the moderating role of psychological security among the relationships of unhappy musicians, resistance to innovation, and uncreative music products in Malaysia. This study has applied the questionnaire method to gather the primary data from the selected respondents. These questionnaires were adapted from past studies, e.g., unhappy musicians (UHM) have been taken as the independent variable with six items taken from Berque et al. (2014), and resistance to innovation has been taken as the independent variable with five items taken from Heidenreich and Handrich (2015). In addition, psychological security (PYS) has been taken as a moderator with six items extracted from Jia et al. (2017). Finally, uncreative music products (UMP) have been taken as a dependent variable with five items taken from Wu et al. (2017). This article has followed the innovation adoption theory that describes that innovation adoption can enhance the quality of the products. This article also examines the role of resistance to innovation on the uncreative music product using the innovation adoption theory that lack of innovation produces uncreative products. Based on this theory, this article has established the framework mentioned in Figure 1.

These questionnaires were distributed to the selected respondents through the mail and personal visits. As major music learning institutions are located in Kuala Lumpur, Malaysia, the students of those institutions are selected based on simple random sampling. The researchers sent about 525 surveys to the selected students, but only 290 were returned. These valid surveys have an approximately 55.24% response rate. In addition, the researchers have also applied the smart-PLS to check the nexus among constructs and test the hypotheses. The smart-PLS is effective software that perfectly executes the larger datasets and complex frameworks (Hair et al., 2021a). Smart-PLS is considered the best statistical tool for the analysis of the primary data (Hair et al., 2021b). It assesses the measurement model for reliability and validity of the constructs and also assesses the structural model for hypotheses testing (Hair et al., 2019).

## Findings of the Research

**FIGURE 2:**
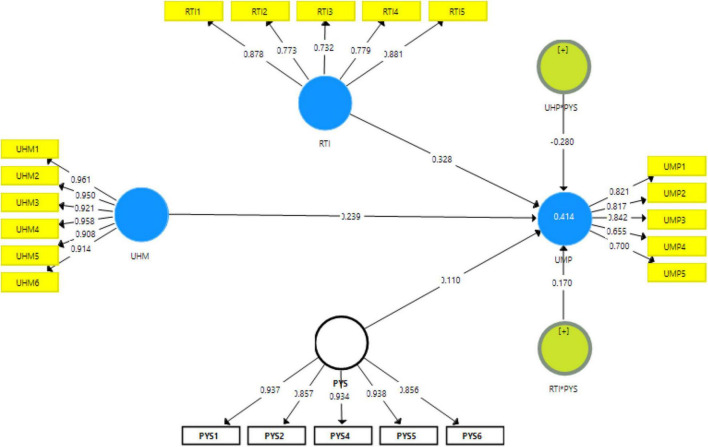
Measurement model assessment.

**FIGURE 3:**
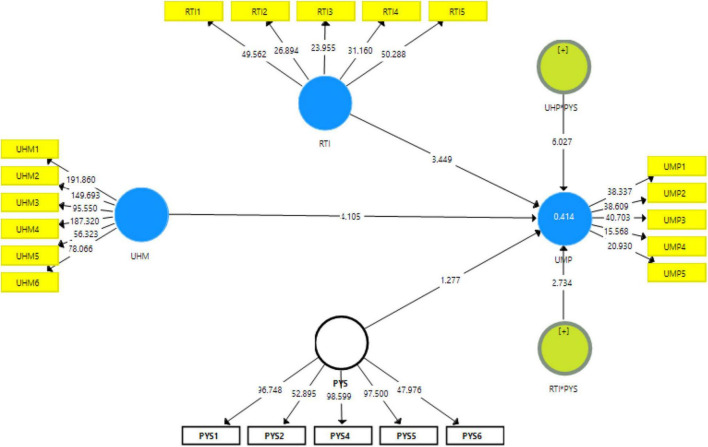
Structural model assessment.

**FIGURE 4:**
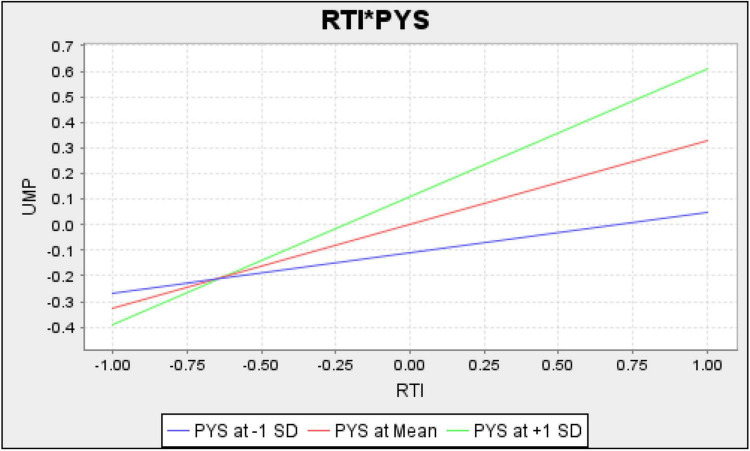
RTI*PHS.

**FIGURE 5:**
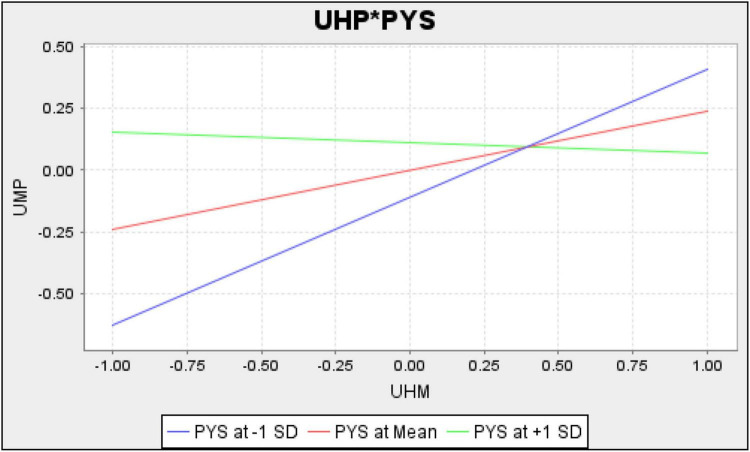
UHP*PHS.

Figure 2 shows measurement model assessment, Figure 3 shows Structural model assessment, Figure 4 shows RTI*PHS and Figure 5 shows results for UHP*PHS respectively.

The results indicated that the correlation among items had been examined using average variance extracted (AVE), and the figures are larger than 0.70. These figures indicated that the convergent validity is valid. In addition, the results indicated that the content validity had been examined using factor loadings, and the figures are larger than 0.50. These figures indicated that the content validity is valid. The results also indicated that the reliability of items had been examined using composite reliability (CR) and alpha, and the figures are larger than 0.70. These figures indicated that the reliability is valid. Table 1 shows the convergent validity of the items.

**TABLE 1:** Convergent validity.

Constructs	Items	Loadings	Alpha	CR	AVE
Psychological Security	PYS1	0.937	0.944	0.958	0.820
	PYS2	0.857			
	PYS4	0.934			
	PYS5	0.938			
	PYS6	0.856			
Resistance toward Innovation	RTI1	0.878	0.868	0.905	0.658
	RTI2	0.773			
	RTI3	0.732			
	RTI4	0.779			
	RTI5	0.881			
Unhappy Musicians	UHM1	0.961	0.971	0.977	0.876
	UHM2	0.950			
	UHM3	0.921			
	UHM4	0.958			
	UHM5	0.908			
	UHM6	0.914			
Uncreative Music Products	UMP1	0.821	0.827	0.879	0.594
	UMP2	0.817			
	UMP3	0.842			
	UMP4	0.655			
	UMP5	0.700			

The results indicated that the correlation among variables had been examined using Fornell Larcker criterion and cross-loadings criterion. The figures revealed that the values that highlighted the relationship with the variable itself are bigger than those that highlighted the relationship with other variables. These values indicated valid discriminant validity. Table 2 and Table 3 show the discriminant validity of the variables.

**TABLE 2:** Fornell Larcker criterion.

	PYS	RTI	UHM	UMP
PYS	0.905			
RTI	0.810	0.811		
UHM	0.499	0.480	0.936	
UMP	0.498	0.505	0.499	0.771

**TABLE 3:** Cross-loadings criterion.

	PYS	RTI	UHM	UMP
PYS1	**0.937**	0.753	0.456	0.455
PYS2	**0.857**	0.706	0.441	0.451
PYS4	**0.934**	0.744	0.459	0.456
PYS5	**0.938**	0.760	0.459	0.443
PYS6	**0.856**	0.703	0.443	0.447
RTI1	0.662	**0.878**	0.387	0.409
RTI2	0.732	**0.773**	0.449	0.449
RTI3	0.541	**0.732**	0.320	0.391
RTI4	0.670	**0.779**	0.398	0.380
RTI5	0.661	**0.881**	0.381	0.406
UHM1	0.461	0.459	**0.961**	0.467
UHM2	0.454	0.466	**0.950**	0.448
UHM3	0.475	0.448	**0.921**	0.459
UHM4	0.466	0.454	**0.958**	0.468
UHM5	0.473	0.429	**0.908**	0.495
UHM6	0.471	0.442	**0.914**	0.458
UMP1	0.491	0.442	0.393	**0.821**
UMP2	0.450	0.463	0.428	**0.817**
UMP3	0.389	0.441	0.401	**0.842**
UMP4	0.285	0.293	0.357	**0.655**
UMP5	0.248	0.253	0.335	**0.700**

*Bold values are main finding.*

The results also indicated the correlation among variables and have been examined using the Heterotrait Monotrait (HTMT) ratio. The figures revealed that the values are not bigger than 0.85. These values indicated valid discriminant validity. Table 4 shows the discriminant validity of the variables.

**TABLE 4:** Heterotrait Monotrait ratio.

	PYS	RTI	UHM	UMP
PYS				
RTI	0.792			
UHM	0.521	0.521		
UMP	0.548	0.579	0.554	

The results revealed that unhappy musicians and resistance to innovation have a significant and positive linkage with uncreative music products in Malaysia and accept H1 and H2 hypotheses. In addition, the results also revealed that the psychological security significantly moderates the linkage among unhappy musicians, resistance to innovation, and uncreative music products in Malaysia and accepts H3 and H4 hypotheses. Table 5 shows the direct and moderating relationships among variables.

**TABLE 5:** A path analysis.

Relationships	Beta	S.D.	T Statistics	*P* Values	L.L.	U.L.
RTI - > UMP	0.328	0.095	3.449	0.000	0.177	0.515
RTI*PYS - > UMP	0.170	0.062	2.734	0.004	0.069	0.269
UHM - > UMP	0.239	0.058	4.105	0.000	0.136	0.327
UHP*PYS - > UMP	−0.280	0.046	6.027	0.000	−0.372	−0.205

## Discussion

The results showed that unhappy musicians are linked to uncreative music products in a positive manner. These results agree with those suggested by Everts et al. (2021), which revealed that the creativity in the music products depends upon the musicians’ emotions that form their thinking and efforts. When the musicians are satisfied with the circumstances and happy in their lives, they have a pleasant mood and think pleasantly about their profession and accessories. While the musicians who are distressed and unhappy with the circumstances of their lives, including personal and professional criteria, feel unpleasant while dealing with the music practices and instruments. The study proves that the unhappy musicians remain uncreative as they have no particular interest. These results also match with those suggested by Morris (2020), which showed that, like in other fields of recreation, in the music world, creativity is an essential element. People want something new and extraordinary in music. The creativity in the musical instruments is helpful to satisfy the people’s wants of creative music, but it depends on the musicians’ creativity, which is the product of their satisfied and happy mood. These results also match with those suggested by Sanders et al. (2021), which showed that musicians have a happy life with respect to their family and profession, and they put all possible efforts to bring creativity to the musical instruments and lyrics.

The results showed that resistance to innovation is linked to uncreative music products positively. These results agree with those suggested by Klement and Strambach (2019), which threw light on the significance of innovation in the music industry. In the music education institutions where innovation practices are being implemented, both the students and tutors practice creativity in the relevant functions. They not only bring creativity in lyrics or music genre but also bring creativity to the musical instruments. But, if there is resistance to innovation, they remain inactive and stick to the traditional sources and instruments to produce music. That is why resistance to innovation keeps the music products uncreative. These results are in line with those suggested by Tran et al. (2018), which examined that in the countries where the people do not want change, newness, or improvement in their recreational lives, particularly in the industry of music, the professional music does not give any special attention to creativity in music processes or instruments in their use. Thus, the lack of urge for innovation causes uncreative music instruments. These results also match with those suggested by Ferraris et al. (2020), which posited that in the institutions that provide music education, there is a tendency to utilize innovative processes and resources utilization, and there is the requirement for creativity in the music instruments. In the institutions where the tutors and musicians have to face resistance in innovation, they cannot follow and implement creativity ideas in music products.

The results also revealed that psychological insecurity is a moderator between unhappy musicians and uncreative music products. These results are in line with those suggested by Albinsson (2018), which highlighted that psychological health matters a lot in the music industry. If the persons involved in the music industry suffer from psychological insecurity such as fear of failure in achieving the goals, fear of losing a relationship or having a linked negative relationship, and confusion about their capability to handle the situation, they remain unsatisfied and unhappy. In case of psychological insecurity, implementing practices for creativity in music practices and the composition becomes almost impossible. Hence, the psychological insecurity in musicians makes the relationship between unhappy musicians and uncreative music products stronger than worse. These results agree with those suggested by Skaggs (2019). In a music education system, where the musicians have to face emotional or psychological anxiety or insecurity, they lose their happiness and satisfaction with their lives, relations, goals, achievement, and profession. Such persons are even unable to follow traditional music practices, so it is possible for them to think about newness in music products. So, psychological insecurity keeps the musicians unhappy with full of anxiety and confusions and makes it impossible for the music product to be creative. In the presence of musicians having psychological insecurity, unhappiness of musicians contributes to uncreative music products.

The results revealed that psychological insecurity moderates resistance to innovation and uncreative music products. These results are in line with those suggested by Saragih et al. (2019), which stated that for the formation and implementation of innovation policies, a sound and watchful mind must be with the management so that changes can be sensed and better response can be given. If persons involved in the management of music education are the victim of psychological insecurity, then they have to face restlessness of mind and cannot form and implement innovation policies. Likewise, musicians or tutors with disturbed minds cannot work for creative music production. So, in case the psychologically insecure musicians are there in the industry, there is resistance to innovation, and there are uncreative music products. These results are also supported by Wachs and Vedres (2021), which showed the significance of psychological health in creative music products. This study implies that the psychologically insecure persons in the music industry do not accept or help implement the innovation policies. The resistance to innovation adoption, either on the part of management, tutors, or musicians, does not allow creativity in the music products.

## Conclusion

The study aimed to examine the contribution of unhappy musicians and resistance to innovation to uncreative music products and determine the psychological insecurity between unhappy musicians, resistance to innovation, and uncreative music products. Authors chose the music industry of China for the collection of data and collected data for unhappy musicians and resistance to innovation, psychological insecurity, and uncreative music product and their relationships through the issuance of questionnaires. The information collected in response to questionnaires showed a positive relationship between unhappy musicians and resistance to innovation to uncreative music products. The results indicated that musicians who are dissatisfied and unsatisfied with their living conditions, including personal and professional criteria, find dealing with music practices and instruments uncomfortable. The study concludes that dissatisfied musicians stay uncreative because they are uninterested in their work. The results also showed that the tutors and musicians in institutions that encounter resistance to innovation are unable to follow and apply creative ideas in music products. The study revealed that psychological insecurity is a moderator between unhappy musicians, resistance to innovation, and uncreative music products. This study implies that the psychological insecurity affects unhappy musicians and resistance to innovation on uncreative music products and strengthens their relationships.

## Implications

This study makes significant contributions to the literature. The study addresses uncreative music products and examines the impacts of unhappy musicians and resistance to innovation on uncreative music products. The unhappy musicians and resistance to innovation are the two different concepts linked to uncreative music products, and these have been discussed separately in the prior literature. This article combines the impacts of unhappy musicians and resistance to innovation on uncreative music products. Moreover, the psychological insecurity has been addressed as a moderator between unhappy musicians, resistance to innovation, and uncreative music products. Only a few studies checked the influences of psychological insecurity on the association between unhappy musicians, resistance to innovation and uncreative music products in the previous literature. So, this study is a great contribution to literature. This study also contributes to the literature on innovation adoption theory that if the institutions resist to adopt innovation, then they fail to produce creative products. The study has great significance in the practical field, especially in the modern world; music and musical instruments have great significance from a social, religious, cultural, and economic perspective. The study guides the government, schools, or academies arranged for music education and provides ways to develop creativity in music by reducing unattractive music products. Thus, this study guides the regulators to develop the regulations to reduce the unhappiness among musicians and motivates the regulators to adopt innovation to increase the creative music product in Malaysia. This article also helps the new researchers in examining this area in the future.

## Limitations and Future Directions

The study guides the music education institutions that try to minimize the unhappiness of musicians and resistance to innovation for reducing uncreative music products. It also guides that the musicians must try to be happy, overcome their psychological insecurity, and reduce their resistance to innovation so that they can encourage creative music products.

Many limitations are associated with this study, and these limitations must be overcome in future literature. This study examines the impacts of only two factors, namely, unhappy musicians and resistance to innovation, as reasons for uncreative music products. Many other factors, such as institutional support, the financial worth of the music academies, and the worth of recreational activities within the country, have great significance to creativity in music products. The use of a limited number of factors as determiners of uncreative music products limits the study scope and application. In future literature, the scope of the study must be given more attention to for improving its validity. To test the validity of the study hypotheses, the authors used the questionnaire distribution technique to collect data for this study. Scholars are supposed to collect data using an empirical, experimental method, and data must be collected over a long period of time in order to produce more credible conclusions. China is the context that authors collected for hypothesis analysis, while in further literature, the analysis must be conducted in multiple countries.

## Data Availability Statement

The original contributions presented in this study are included in the article/supplementary material, further inquiries can be directed to the corresponding author.

## Author Contributions

WL contributed in construct introduction, literature, methodology and analysis. SA contributed in conclusion. TK contributed in proofreading and reviewed the whole article. All authors contributed to the article and approved the submitted version.

## Conflict of Interest

The authors declare that the research was conducted in the absence of any commercial or financial relationships that could be construed as a potential conflict of interest.

## Publisher’s Note

All claims expressed in this article are solely those of the authors and do not necessarily represent those of their affiliated organizations, or those of the publisher, the editors and the reviewers. Any product that may be evaluated in this article, or claim that may be made by its manufacturer, is not guaranteed or endorsed by the publisher.
